# Informing theoretical development of salutogenic, asset-based health improvement to reduce syndemics among gay, bisexual and other men who have sex with men: Empirical evidence from secondary analysis of multi-national, online cross-sectional surveys

**DOI:** 10.1016/j.ssmph.2019.100519

**Published:** 2019-11-27

**Authors:** Lisa M. McDaid, Paul Flowers, Olivier Ferlatte, Kareena McAloney-Kocaman, Mark Gilbert, Jamie Frankis

**Affiliations:** a MRC/CSO Social and Public Health Sciences Unit, University of Glasgow, 200 Renfield Street, Glasgow, G2 3QB, UK; b Department of Social and Preventative Medicine, School of Public Health, University of Montreal, 7101 Avenue du Parc (3rd Floor), Montreal, Quebec, H3N 1X9, Canada; c Community Based Research Centre for Gay Men's Health, 1007-808 Nelson Street, Vancouver, British Columbia, V6Z 2H2, Canada; d Department of Psychology, Glasgow Caledonian University, Cowcaddens Road, Glasgow, G4 0BA, UK; e School of Population and Public Health, University of British Columbia, 2206 East Mall, Vancouver, British Columbia, V6T 1Z3, Canada; f Department of Health & Community Sciences, School of Health and Life Sciences, Glasgow Caledonian University, Cowcaddens Road, Glasgow, G4 0BA, UK

**Keywords:** Syndemics, Salutogenesis, Multimorbidities, Assets, Men who have sex with men, Sexual health, Mental health

## Abstract

Globally, gay, bisexual and other men who have sex with men (GBMSM) experience an increased burden of poor sexual, mental and physical health. Syndemics theory provides a framework to understand comorbidities and health among marginalised populations. Syndemics theory attempts to account for the social, environmental, and other structural contexts that are driving and/or sustaining simultaneous multiple negative health outcomes, but has been widely critiqued. In this paper, we conceptualise a new framework to counter syndemics by assessing the key theoretical mechanisms by which pathogenic social context variables relate to ill-health. Subsequently, we examine how salutogenic, assets-based approaches to health improvement could function among GBMSM across diverse national contexts. Comparative quantitative secondary analysis of data on syndemics and community assets are presented from two international, online, cross-sectional surveys of GBMSM (SMMASH2 in Scotland, Wales, Northern Ireland and the Republic of Ireland and Sex Now in Canada). Negative sexual, mental and physical health outcomes were clustered as hypothesised, providing evidence of the syndemic. We found that syndemic ill-health was associated with social isolation and the experience of stigma and discrimination, but this varied across national contexts. Moreover, while some of our measures of community assets appeared to have a protective effect on syndemic ill-health, others did not. These results present an important step forward in our understanding of syndemic ill-health and provide new insights into how to intervene to reduce it. They point to a theoretical mechanism through which salutogenic approaches to health improvement could function and provide new strategies for working with communities to understand the proposed processes of change that are required. To move forward, we suggest conceptualising syndemics within a complex adaptive systems model, which enables consideration of the development, sustainment and resilience to syndemics both within individuals and at the population-level.

## Highlights

• Gay and other men who have sex with men experience syndemics in varied contexts.
• Some salutogenic community assets have a protective effect on syndemic ill-health.
• Conceptualising syndemics within a complex adaptive systems model is required.

## Introduction

Globally, gay, bisexual and other men who have sex with men (henceforth GBMSM) experience an increased burden of poor sexual, mental and physical health when compared to their heterosexual counterparts (Beyrer et al., 2016; Stall et al., 2016). Marked inequalities in mental health have been reported in multiple studies, with gay and bisexual (GB) populations found to be at greater risk of suicide, mood disorders, and anxiety than heterosexual populations (King et al., 2008), and higher prevalence of acute and chronic physical conditions and illnesses has also been reported (Lick, Durso, & Johnson, 2013; Mercer et al., 2016; Sandfort, Bakker, Schellevis, & Vanwesenbeeck, 2006). That the experience of poor sexual, mental and physical health could be interrelated is of concern. For example, in Great Britain's third National Survey of Sexual Attitudes and Lifestyles (Natsal-3), 8.4% of MSM reported experiencing simultaneously sexual, mental and physical health problems, compared with just 1.5% of men who have sex exclusively with women reporting all three (Mercer et al., 2016). Understanding how and why such negative health outcomes cluster, and what we can do to intervene to prevent this, is a matter of urgency for global public health.

‘Syndemics’ is the term given to the co-occurring and co-reinforcing multiple, interrelated health conditions, which develop and are sustained due to harmful social contexts (Singer & Clair, 2003). The concept, first proposed in the late 1990s, has been suggested as a framework for understanding health among marginalised populations, such as GBMSM, which can account for the social, environmental, and other structural contexts that are driving and/or sustaining negative outcomes. A 2017 Lancet series emphasised the importance of accounting for the latter, building on traditional medical approaches to co- and multi-morbidities (Singer, Bulled, Ostrach, & Mendenhall, 2017).

In so doing, the focus is on the *upstream*, distal, shared and overlapping determinants of multiple pathologies (e.g., the ongoing impact of legislation relating to the criminalisation of homosexuality, the deep and insidious impact of heteronormativity, homophobic bullying in educational settings, homophobic microaggressions within the workplace). This contrasts with the typical *downstream* approach, examining the proximal determinants of single behaviours, one at a time (e.g., low self-efficacy in relation to condom use) or indeed understanding and moderating socially embedded structural determinants of ill-health through an individual lens alone (e.g., minority stress theory (Pachankis, 2015)).

How to address the combined impact of social and structural determinants on health and how to develop and deliver upstream interventions to counter these become the core questions for public health (Willen, Knipper, Abadía-Barrero, & Davidovitch, 2017). A syndemic approach to understanding health inequities among GBMSM is particularly pertinent since it grew out of efforts to understand and counter the HIV/AIDS epidemic (Singer et al., 2017), and has since been applied to understanding the co-occurring syndemic drivers of other sexually transmitted infections (e.g., syphilis) (Ferlatte et al., 2018), suicide (Ferlatte, Dulai, Hottes, Trussler, & Marchand, 2015; Mustanski, Andrews, Herrick, Stall, & Schnarrs, 2014) and smoking (Storholm, Halkitis, Siconolfi, & Moeller, 2011).

Syndemics theory is not without methodological critique, particularly centering on the lack of empirical evidence for the extent to which health conditions interact (Tsai & Burns, 2015; Tsai, Mendenhall, Trostle, & Kawachi, 2017). Indeed, most syndemic studies have only presented correlations and associations between sexual health outcomes and/or examined whether groupings of these have a cumulative effect on sexual risk behaviour or HIV as the primary outcome. Meanwhile, studies that have provided evidence of disease interactions have been rarer (Tsai & Burns, 2015). Tsai et al. have also noted that while syndemics theory suggests disease interaction operates at the individual- and population-level, empirical literature includes only individual-level outcome and covariate data (and none have included ecological, multilevel or experimental study designs) (Tsai et al., 2017).

A second critique can be made of the impoverished operationalisation of social contexts within syndemics theory. While it is recognised that syndemic ill-health is produced by social inequities and marginalization the literature examining how syndemics among GBMSM are driven by negative and pathogenic social contexts at the population-level is limited (e.g., the experience of homophobia, stigma and discrimination, and social isolation as evidence of potential distal, upstream determinants). Only a few exceptions have measured these social contextual factors (see e.g., (Mustanski et al., 2014); (Ferlatte et al., 2014, 2015, 2018; Ostrach, Lerman, & Singer, 2017).

How to intervene to reduce syndemics is also unclear (Tsai et al., 2017). By positing that multiple interrelated health inequalities among GBMSM are grounded in, and driven by, the pathogenic social contexts in which they live and the synergistic accumulation of psychosocial problems and ill health, it has been suggested that improving these outcomes will require interventions designed to have a synergistic, salutogenic effect at multiple levels (Stall, Coulter, Friedman, & Plankey, 2015). Salutogenesis is broadly defined as having a focus on the positive, on health, as opposed to ill-health and disease, and aims to understand what are the strengths and assets that we can draw on to improve health (Mittelmark et al., 2017). Assets are the collective resources available for the promotion of health and wellbeing, and interventions focus on enabling salutogenic, protective factors and resources of individuals, families, communities and organisations, rather than deficits and needs (Antonovsky, 1996; Morgan & Ziglio, 2007). Asset-based approaches are forms of engagement and relationship building, underpinned by values and principles, which enable strengths, capacities and abilities to be identified and developed for positive outcomes, and sustained through people's actions, connections and participation. Health assets operate across multiple levels, for example in self-esteem and resilience at the individual-level, supportive friendship and peer networks at families of choice and community-levels, and provision of positive environmental and organisational resources to promote health and wellbeing at the structural-level (Morgan & Ziglio, 2007). While evidence of effectiveness of asset-based approaches is still limited (Morgan & Ziglio, 2007), and largely absent from health improvement efforts with GBMSM so far, there have been calls to develop such efforts to reduce their multiple, interrelated health inequalities (Halkitis, 2013; Halkitis, Krause, & Vieira, 2017; Herrick et al., 2011, 2014; Mayer et al., 2012). Asset or strengths and resilience approaches have been suggested as a means of countering syndemics (Chakrapani, Kaur, Newman, Mittal, & Kumar, 2019; Herrick et al., 2011; Stall, Friedman, & Catania, 2008), but the approach has not been fully defined, or tested, with studies again focussing on the unitary outcomes of HIV, sexual risk behaviour or substance use (Adeboye et al., 2017; Chakrapani, Newman, Shunmugam, Logie, & Samuel, 2017; Chakrapani et al., 2019; Cleland, Lanza, Vasilenko, & Gwadz, 2017; Halkitis et al., 2017; Hart et al., 2017; McNair et al., 2018).

The need to intervene simultaneously at the individual-, network- and community-level to address syndemics has also been contested and there is no evidence to demonstrate that interventions focused on a single issue might not be effective (Tsai et al., 2017). Conceptualising such health inequalities within a complex adaptive systems model suggests health inequalities derive from multiple interrelated factors operating within a connected whole and it takes account of the social context within which people live, advocating that, rather than seeking solutions to individual problems, we should focus on how to change aspects of the system to promote positive change within it (Rutter et al., 2017). This is very much in line with the approaches promoted within the existing syndemics literature (Stall et al., 2015; Herrick et al., 2011).

This paper makes a major contribution to the syndemics literature by providing further international evidence of the clustering of ill-health amongst populations of GBMSM. Moving beyond this empirical contribution, we address criticism of syndemics theory and begin to explore the potential theoretical mechanisms that can explain and moderate the upstream determinants of ill-health. To conceptualise a new framework for interventions to counter syndemics, we must understand and test out the key theoretical mechanisms by which pathogenic social context variables relate to ill-health and also examine how salutogenic, asset-based approaches to health improvements could function to improve health. We test three hypotheses:

1. Negative sexual, mental and physical health outcomes will cluster providing evidence of the syndemic
2. Syndemic ill-health will vary significantly according to pathogenic social context (i.e., the experience of stigma and discrimination and social isolation)
3. Salutogenic, community assets will have a protective effect on syndemic ill-health

## Methods

### Data sources

Comparative quantitative secondary analysis of data on syndemics and community assets are presented from two international, online, cross-sectional surveys of GBMSM. Use of the two surveys enabled examination of a range of survey measures that were hypothesised to contribute to syndemics and community assets, while also accounting for different social contexts (noted above as important for the development and evaluation of complex interventions).

### Study design

SMMASH2 (a study of social media, sexual and holistic health) collected anonymous, self-complete questionnaires online from April to June 2016 in Scotland, Wales, Northern Ireland (NI) and the Republic of Ireland (RoI). Men who accessed GBMSM-specific social media websites and apps (Gaydar, Recon, Grindr, Growlr, Squirt and Hornet) were invited to participate either via message blasts or banner advertisements. Ethical approval was granted by Glasgow Caledonian University School of Health and Life Sciences Ethics Subcommittee (HLS id: HLS/NCH/15/26) and consent assumed by survey participation.

Sex Now is a community-based serial survey of Canadian GBMSM's health, sex and daily lives. Data for this article derived from the 2015 edition, which was collected from November 2014 to 2015. GBMSM were recruited through online promotion within sex-seeking websites (Squirt, Fridae, Daddyhunt) and apps (Grindr and scruff), community groups, gay media, social media, and e-mail to participants from previous editions. Surveys were completed anonymously online, in one of Canada's two official languages (English or French). The independent ethics board of the Community-Based Research Centre for Gay Men's Health reviewed and approved this project and participants indicated informed consent by clicking on a button before accessing the survey questions.

### Participants

SMMASH2: 3220 GBMSM completed questionnaires; Scotland (n = 1551); Wales (n = 509); NI (n = 244); and RoI (n = 916). An initial effective sample of 2971 men for analysis of syndemics was identified based on valid responses to the health outcomes variables which contribute to the outcome of interest (Scotland, n = 1452; Wales, n = 471; NI, n = 229; and RoI, n = 819). The sample was further restricted for regression analysis, based on valid response to all items identified as significant in bivariate analysis, leaving a sample of 1715 for this analytic stage (Scotland, n = 868; Wales, n = 274; NI, n = 130; and RoI, n = 443). Comparisons of the valid sample for regression analysis with those excluded from that analysis revealed no significant differences in the presence of syndemic health outcomes (χ^2^_(1)_ = 2.715, p = .099).

Sex Now: 742 MSM completed the survey in French and 7130 in English for a total of 7872 respondents. To assess the reach of the survey, respondents were asked to provide the first three digits of their postal code. Known as Forward Sorting Area (FSA), these digits helped link participants to their town or region within Canada. Surveys were received from 1362 FSAs out of 1620, representing an 84% coverage. Participation in the survey was voluntary and participants could decide not to answer or stop their participation at any time. Only fully completed surveys were collected for research purposes, as such, there are no missing data for the study and all 7872 cases were included in the analysis.

### Measures

Study measures in SMMASH2 and Sex Now included: sociodemographics; a range of sexual, mental and physical ill-health outcomes, along with their hypothesised contextual determinants; experience of stigma and discrimination and social isolation (as measures of pathogenic context); and a range of measures of salutogenic community assets (sense of coherence and emotional competence, aspirations, and community engagement). The survey measures were comparable in terms of topics, but used different question wording, and at times different response options. For this reason, we did not combine the data and instead conducted separate analyses of data from the two surveys. Online Supplementary File 1 provides further details of the scales included, the specific wording of questions and references to underpin their psychometric properties, where appropriate.

Syndemic ill-health: This was derived to record the presence of a sexual, physical and mental ill-health outcome (see online supplementary file 1 for the specific variables that underpinned sexual, physical and mental ill-health outcomes, respectively). A syndemic ill-health outcome variable was then derived to reflect the experience of 2 or more sexual, mental and physical health outcomes by an individual (and compare this to participants reporting one or none, given that someone with a single outcome is not experiencing the ‘syndemic’). An additional variable was created to reflect the presence of behaviours that could be considered drivers for the outcomes using the same approach as above, coded for none, sexual only, physical only, and both sexual and physical health behaviours (see supplementary file 1 for the specific variables that underpinned these drivers).

### Analysis

Data were analysed with IBM SPSS 23, and Excel. In order to investigate statistical dependence of the three ill-health outcomes, a cluster analysis using the observed/expected (O/E) ratio method was most appropriate, given the small number of outcomes of interest (Hardy et al., 2012). The O/E ratio method provides an indication of the statistical interdependence of the separate outcomes. Outcomes are clustered when the observed frequency of the combination differs from that expected if the outcomes are independent. O/E ratio values greater than 1.00 indicate higher prevalence than expected if independent, values of 1.00 indicate prevalence in line with independence of the variables; and values from 0.00 to 0.99 indicate prevalence lower than expected. 95% confidence intervals are produced as an indication of the significance of the clustering. O/E ratio calculations and bivariate analyses were performed with data from all GBMSM with valid date on the relevant outcomes, to maximise the sample available to make determinations of clustering.

Chi square and independent samples t-tests were performed to identify significant bivariate relationships for further exploration in regression models. Variables significant at the bivariate-level (p < .05) were entered into a hierarchical logistic regression model used to estimate odds ratios and 95% confidence intervals of syndemic ill-health, with sociodemographics entered at the first step, social isolation variables included at the second step, and community assets variables included at the third step. For the SMMASH2 regression analysis, a complete case analysis was performed with a reduced sample due to missing data on the variables of interest identified as significant at the bivariate-level (n = 1715).

## Results

### Sample characteristics

In SMMASH 2 the highest proportion of participants were in the ≥46 years age group (37%, n = 1102; 35–45 = 23%, n = 693; 26–35 = 23%, n = 559; 16–25 = 17%, n = 500). The sample was predominately white (97%, n = 2879), employed (74%) and degree level education or higher (62%). The majority of the SMMASH2 sample identified as gay (81%, n = 2380), and half (51%, n = 1387) reported having had a recent HIV test (within previous 12 months). In Sex Now the highest proportion of individuals were in the >=46 years age group (46%, n = 3582; 36–45 = 18%, n = 1358; 26–35 = 22%, n = 1719; 18–25 = 15%, n = 1157). The sample was predominately white (81%, n = 6365), employed (91%, n = 7148) and degree level education or higher (64%, n = 4997). The majority of the Sex Now sample identified as gay (65%, n = 5062), and 16% (n = 1266) reported not having had an HIV test.

### Co-occurrence and clustering of syndemic ill health outcomes

Hypothesis 1 Negative sexual, mental and physical health outcomes will cluster providing evidence of the syndemic.

The prevalence of each individual ill-health outcome is shown in Online Supplementary File 2. The O/E ratio cluster analysis indicated that the three negative ill-health outcomes are clustered with statistical interdependence among the outcomes (Table 1). Having none of the three outcomes was almost three times more prevalent than expected in SMMASH2; the rules of probability give an expected prevalence of 3.2% of the sample having none of these outcomes if they were independent, in comparison to the observed prevalence of 9.8% of the sample. Similarly having none of the outcomes was 1.2 times more prevalent than expected in Sex Now; here the rules of probability indicated an expected prevalence of 27.1% when the observed prevalence was 31.2% of the sample reporting none of the outcomes. The presence of all three outcomes was 1.3 times (in SMMASH2) and 1.6 times (in Sex Now) more prevalent than expected if the outcomes were independent. In both samples, the presence of sexual or mental ill-health outcomes in isolation was significantly less prevalent than expected, as was the occurrence of any pair of outcomes in SMMASH2. In Sex Now, the presence of mental and physical ill-health outcomes was not significant, and the presence of sexual and mental ill-health outcomes was 1.2 times more prevalent than expected.

**Table 1:** Clustering of syndemic health outcomes indicated by Observed/Expected (O/E) ratio and 95% confidence intervals (CI).

	SMMASH2			Sex Now		
	Prevalence %	O/E ratio	95% CI	Prevalence %	O/E ratio	95% CI
No health outcomes	**9.80**	**2.93**	**2.58–3.29**	**31.20**	**1.15**	**1.10–1.19**
Sex only	**10.70**	**0.88**	**0.78–0.97**	**7.40**	**0.85**	**0.77–0.91**
Mental only	**2.30**	**0.45**	**0.34–0.56**	**14.80**	**0.80**	**0.75–0.85**
Physical only	5.30	1.10	0.93–1.28	17.10	0.96	0.91–1.01
Sex and Mental	**17.20**	**0.88**	**0.81–0.96**	**7.00**	**1.18**	**1.08–1.28**
Sex and Physical	**12.60**	**0.69**	**0.62–0.76**	**3.80**	**0.66**	**0.60–0.73**
Mental and physical	**3.40**	**0.44**	**0.36–0.53**	12.60	1.04	0.97–1.10
All three	**38.70**	**1.32**	**1.25–1.40**	**6.20**	**1.59**	**1.45–1.73**

Notes: Bold text indicates significant clusters.

In summary, negative sexual, mental and physical health outcomes were clustered, providing evidence of the syndemic.

### Sociodemographic and pathogenic associations with syndemic ill-health

Hypothesis 2 Syndemic ill-health will vary significantly according to pathogenic social context (i.e., the experience of stigma and discrimination and social isolation)

. Sociodemographic associations with syndemic ill-health are shown in Online Supplementary File 3. In both samples, the social isolation variable of self-reported ‘outness’ was associated with syndemic ill-health. For the other social isolation variables, in SMMASH2, preferred relationship status was significant, while the experience of stigma was not. In Sex Now, the four measures of satisfaction with meeting partners were significantly associated with syndemic ill-health, as was the experience of discrimination.

The hierarchical logistic regression models are shown in Table 2. When the social isolation variables were added at step 2, all significant sociodemographic associations remained, and at a similar magnitude, but neither social isolation variable was significantly associated with syndemic ill-health in the SMMASH2 sample. In Sex Now, the sociodemographics also remained significantly associated with syndemic ill-health, as did social isolation variables of outness, experience of discrimination, worry about stigma, and dissatisfaction with meeting partners at social events and bars.

**Table 2:** Hierarchical logistic regression models of syndemic ill health (the experience of 2 or more negative health outcomes) among participants in the SMMASH2 and Sex Now surveys: Odds ratios (OR) and 95% Confidence Intervals (CI).

	SMMASH2						Sex Now					
	Syndemic ill health						Syndemic ill health					
	Step 1		Step 2		Step 3		Step 1		Step 2		Step 3	
	**OR**	**95%CI**	**OR**	**95%CI**	**OR**	**95%CI**	**OR**	**95%CI**	**OR**	**95%CI**	**OR**	**95%CI**
***Socio-demographics***												
**Age**												
18–25							1		1		1	
26–35							**1.30**	**1.07–1.58**	**1.31**	**1.08–1.59**	**1.19**	**0.98–1.46**
36–45							**1.91**	**1.55–2.34**	**2.10**	**1.71–2.59**	**1.80**	**1.45–2.23**
46 +							**2.28**	**1.90–2.74**	**2.74**	**2.27–3.32**	**2.31**	**1.90–2.81**
**Highest Qualification**												
None	1		1		1							
Secondary	0.36	0.05–2.97	0.36	0.04–2.96	0.43	0.05–3.47						
Degree	0.32	0.04–2.62	0.32	0.04–2.62	0.39	0.05–3.13						
Postgraduate	0.33	0.04–2.72	0.33	0.04–2.73	0.40	0.05–3.29						
**Orientation**												
Gay	1		1		1		1		1		1	
Bisexual	0.91	0.56–1.49	0.85	0.51–1.43	0.82	0.49–1.39	0.92	0.79–1.09	1.11	0.93–1.32	1.06	0.88–1.27
Straight (+others for Sex Now)	0.38	0.06–2.36	0.35	0.06–2.21	0.34	0.05–2.18	1.60	0.91–1.25	1.08	0.92–1.28	1.02	0.87–1.21
**Relationship Status**												
Single	1		1		1		1		1		1	
Regular Male Partner	0.98	0.69–1.41	0.98	0.67–1.43	0.94	0.64–1.38						
CP/Married	0.79	0.49–1.29	0.76	0.42–1.35	0.75	0.42–1.35						
Regular female partner	1.41	0.76–2.62	0.25	0.65–2.45	1.36	0.70–2.64						
Partnered with a man							**0.80**	**0.71–0.90**	**0.81**	**0.71–0.92**	0.86	0.69–1.02
Partnered with a woman							**0.66**	**0.55–0.79**	**0.80**	**0.66–0.97**	0.84	0.69–1.02
Separated, widowed, Other							1.14	0.95–1.38	1.16	0.96–1.41	1.16	0.95–1.41
**Employment Status**												
Employed	1		1		1		1		1		1	
Unemployed	1.03	0.43–2.45	1.03	0.43–2.45	0.95	0.40–2.27	**1.97**	**1.70–2.34**	**1.91**	**1.61–2.28**	**1.68**	**1.40–2.01**
Retired	1.40	0.82–2.39	1.38	0.81–2.37	1.49	0.87–2.58						
Student	0.59	0.35–1.02	0.59	0.34–1.01	**0.54**	**0.31–0.93**						
Long-term sick/carer	2.44	0.55–10.72	2.42	0.55–10.65	2.10	0.47–9.33						
**Financial Worries**												
Never/occasionally	1		1		1							
Sometimes/Always	**1.74**	**1.23–2.47**	**1.75**	**1.24–2.48**	**1.64**	**1.15–2.33**						
**Ethnicity**												
White							1		1		1	
Indigenous							**1.45**	**1.15–1.83**	**1.41**	**1.11–1.79**	**1.47**	**1.15–1.86**
Non-white							1.11	0.96–1.29	1.08	0.93–1.25	**1.11**	**1.15–1.86**
**HIV status**												
HIV positive							1		1		1	
HIV negative							**0.66**	**0.55–0.78**	**0.66**	**0.55–0.78**	**0.66**	**0.55–0.79**
Untested							**0.47**	**0.37–0.59**	**0.50**	**0.40–0.63**	**0.48**	**0.38–0.60**
**Income**												
>60,000							1		1		1	
30,000–59,999							1.10	0.97–1.24	1.11	0.98–1.26	1.00	0.87–1.13
<30,000							**1.44**	**1.25–1.67**	**1.43**	**1.23–1.65**	**1.17**	**1.00–1.37**
***Syndemic drivers***												
None	1		1		1		1		1		1	
Sexual only	0.98	0.70–1.36	0.98	0.70–1.36	0.98	0.70–1.37	**1.64**	**1.12–2.40**	**1.57**	**1.07–2.32**	**1.53**	**1.04–2.26**
Physical only	**11.57**	**6.59–19.26**	**11.71**	**7.03–19.52**	**8.66**	**5.08–14.76**	**1.57**	**1.37–1.79**	**1.53**	**1.34–1.76**	**1.50**	**1.31–1.72**
Both	**24.65**	**10.22–54.74**	**24.06**	**10.38–55.76**	**17.21**	**7.30–40.56**	**2.11**	**1.75–2.53**	**2.04**	**1.69–2.47**	**1.96**	**1.62–2.37**
***Social isolation***												
**Preferred relationship status**												
Current			1		1							
Different			0.90	0.63–1.30	0.89	0.61–1.28						
Don't mind			1.24	0.33–4.71	1.00	0.26–3.82						
Outness			0.96	0.84–1.09	0.97	0.85–1.11						
**Dissatisfied with meeting men**												
Social									**1.15**	**1.01–1.32**	1.06	0.93–1.21
Bar									**1.25**	**1.09–1.43**	**1.24**	**1.08–1.42**
Internet									1.08	0.96–1.22	1.05	0.93–1.20
Apps									1.08	0.95–1.23	1.05	0.92–1.19
**Experience of discrimination in the last 12 months**									**1.87**	**1.63–2.14**	**1.83**	**1.60–2.10**
**Worry of stigma**									**1.45**	**1.30–1.62**	**1.87**	**1.23–1.54**
**Out to everyone**									**1.24**	**1.08–1.43**	**1.28**	**1.10–1.47**
***Assets***												
**Sense of coherence**					**0.98**	**0.96–0.998**						
**Emotional competence**					1.11	0.85–1.45						
**Aspirations**												
Unlikely to achieve desired quality of life											**1.89**	**1.63–2.19**
Unlikely to have enough money to live as you wish											**1.20**	**1.03–1.38**
Unlikely to own property											**1.16**	**1.01–1.32**

Notes: Bold text indicates significant Odds Ratios.

In summary, syndemic ill-health varied significantly according to pathogenic social context (i.e., the experience of stigma and discrimination and social isolation) in the Sex Now survey only.

### Community assets and syndemic ill-health

Hypothesis 3 Salutogenic community assets will have a protective effect on syndemic ill-health.

The community assets variables, sense of coherence and emotional competence, were significantly associated with syndemic ill-health in SMMASH2, as were the aspirations variables in Sex Now, but community engagement was not (Online Supplementary File 3).

In the final step of the logistic regression models, sense of coherence was significant in SMMASH2 (Table 2), indicating that higher sense of coherence was associated with decreased odds of syndemic ill-health. Financial worries and syndemic drivers also remained significantly associated with syndemic ill-health, but to a lower magnitude than when the assets variables were not considered. A significant relationship emerges with employment status, with students less likely to report syndemic ill-health in comparison to the employed.

In Sex Now, when the assets variables were added to the hierarchical regression model, aspirations were significant, with being unlikely to achieve quality of life, being unlikely to have enough money to live as you wish, and being unlikely to own property being associated with syndemic ill-health. All of the sociodemographics, other than relationship status, and syndemic drivers remained significantly associated with higher odds of syndemic ill-health, as were the social isolation variables of outness, experience of discrimination, worry about stigma, and dissatisfaction with meeting partners at bars.

In summary, our hypothesis is partly supported. Analyses suggest that some salutogenic community assets (sense of coherence and aspirations) could have a protective effect on syndemic ill-health, while others did not (emotional competency and community engagement).

## Discussion

Our study has confirmed the presence of concurrent, sexual, physical and mental ill-health in communities of GBMSM across five high income countries and, despite considerable variation in their extent, significant clustering of these. We found that syndemic ill-health was associated with social isolation and the experience of stigma and discrimination, but varied across national context. Some of our measures of community assets appeared to have a protective effect on syndemic ill-health, while others did not. Moreover, absence of ill-health also clustered together, providing further evidence that negative health outcomes serve to make others more likely. These results present an important step forward in our understanding of syndemics and provide new insights into how to intervene to reduce these. Below, we summarise and reflect on our mixed evidence, the strengths and weaknesses of our study, and then discuss the implications for further research it suggests (Table 3).

**Table 3:** Summary of evidence, comparison and future policy, practice and research implications.

	Evidence from SMMASH2	Evidence from Sex Now	Comparison, interpretation and implications
Co-occurrence and clustering of syndemic ill-health outcomes	Strong	Some	International evidence, although differentially realised, suggestive of macrosocial structural determinants of syndemic ill-health: • Health improvement efforts with GBMSM should look beyond sexual health and HIV • Behavioural surveillance across countries should be harmonised to strengthen research • International comparative analyses should compare macro-social pathogenic and salutogenic factors and their relation to syndemic ill-health
The role of pathogenic social context in shaping syndemic ill-health	No evidence for stigma, discrimination and social isolation (once individual health behaviours controlled for), but strong evidence for the role of financial hardship and austerity	Strong evidence of stigma, discrimination and social isolation	International evidence highlights the role of pathogenic social contexts in relation to syndemic ill-health but these are realised differently across the studies: • The experience of stigma and discrimination may not be as pertinent, as financial hardship is in a time of ongoing austerity • Individual health behaviours should be conceptualised as intermediate steps in the casual chain by which syndemics affect health; they should not be considered as primary outcomes, • Need to moderate macro-social elements that distinguish the national contexts and assess which structural elements are amenable to change • Need for further research using less individualised methods and multi-level models encapsulating new and mixed methods approaches
The role for the salutogenic context in moderating syndemic ill-health	Strong evidence for sense of coherence, but not emotional competency	Strong evidence for aspirations, but not community engagement	International evidence of the role of salutogenic social context but realised differently across the studies: • The protective effect of sense of coherence and aspirations demonstrates a theoretical mechanism by which salutogenic, asset based approaches could function • Empirical, formative research is required to map out and understand social relationships and networks and their supra-individual role in moderating the determinants of syndemic-ill health -this will be vital for future intervention development. • Need to conceptualise health inequalities within a complex adaptive systems model, which enables consideration of syndemics within individuals and how population-level factors could increase risk or foster resilience over time • Enact a combined approach to encapsulate the preventative complex interventions alongside stratified, targeted and intensive reparative interventions for those most affected by syndemics

### Negative sexual, mental and physical health outcomes will cluster providing evidence of the syndemic

Syndemics analyses have been critiqued for not fully investigating how each component is related to the other (Tsai & Burns, 2015; Tsai et al., 2017), and we have provided evidence of concurrent and clustered negative sexual, mental and physical health outcomes. Our study differs from much of the previous literature, which has mainly focused on associations between mental health and individual psychosocial health behaviours that make up the syndemic, and the effect of these on sexual risk behaviour or HIV as the primary outcome. That substantial proportions of MSM in SMMASH2 and Sex Now reported experience of all three negative mental, physical and sexual health outcomes suggests an unmet need for health improvement that goes beyond anything our new (albeit most welcomed) biomedical interventions (i.e., PrEP) can provide (McCormack et al., 2016). Unless we also address the varied disparities and inequalities in health outlined here, the promise of these biomedical solutions cannot be realised. Our study contributes to a growing body of international literature endorsing the need for health improvement efforts with GBMSM to look beyond sexual health and HIV (Halkitis, Kapadia, Ompad, & Perez-Figueroa, 2015) with recent interventions targeting holistic issues including anxiety/depression (Pachankis, Hatzenbuehler, Rendina, Safren, & Parsons, 2015), LGBT affirmative therapy (O'Shaughnessy & Speir, 2018), problematic alcohol use (Wray et al., 2016), mindfulness (Iacono, 2019), gay-straight alliances (Marx & Kettrey, 2016) and reducing homophobia (Bartos & Hegarty, 2019), operating at micro, meso- and macro sociocultural levels.

### Syndemic ill-health will vary significantly according to the experience of stigma and discrimination and social isolation

In contrast to much of the previous literature, our study has more thoroughly examined the potential association of the pathogenic social context within which GBMSM live and syndemics. Our hypothesis that syndemic ill-health would vary significantly according to the experience of stigma and discrimination and social isolation was upheld in the Sex Now data, but not once other socio-demographics and syndemic drivers (e.g. individual health behaviours) were controlled for in the SMMASH2 data. Instead, financial hardship and physical and sexual health drivers were the factors significantly associated with syndemic ill-health. This is of course not surprising, given our knowledge of these as more proximal individual-level risk factors for poor ill-health (Jepson, Harris, Platt, & Tannahill, 2010). However, as Tsai et al. have suggested, we should not conclude that these individual risk factors are the primary cause of syndemic ill-health (Tsai et al., 2017). These behaviours may be as much a symptom of the experience of syndemics as a cause and indeed we would argue that they are as much a factor of the pathogenic social context, as stigma and discrimination are. Furthermore, differences between SMMASH2 and Sex Now point to the need to consider the local setting in our understanding of social context.

### Community assets will have a protective effect on syndemic ill-health

Given that syndemic health inequalities are proposed to be grounded in and driven by negative and pathogenic social contexts, it has been suggested that countering this could require a salutogenic, asset-based approach for health improvement (Brooks & Kendall, 2013; Chakrapani et al., 2019; Durie & Wyatt, 2013; Herrick et al., 2011; Stall et al., 2008). The protective effect of sense of coherence and aspirations demonstrates a theoretical mechanism by which salutogenic, asset-based approaches to health improvement could function. Previous research has examined the protective effect of resilience on individual outcomes, such as HIV (Adeboye et al., 2017; Chakrapani et al., 2017, 2019; Cleland et al., 2017; Hart et al., 2017; McNair et al., 2018; Woodward, Banks, Marks, & Pantalone, 2017), and our study provides empirical evidence that capitalising on and promoting such community assets could indeed be a means of countering syndemics. It points to the need for an integrated approach; capitalising on collective assets at the organisational-, community- and individual-level. However, sense of coherence could be argued to measure just one, limited form of resilience within a community (and one that is primarily individual). The lack of association between community engagement and syndemic ill-health in our Canadian sample suggests that our attempts to measure this are perhaps too simplistic and inadequate, or we do not yet fully understand the full range of collective assets that could be drawn upon. Indeed, the very concept of resilience has been criticised as not being cognisant of the unique experiences of sexual minority communities (Colpitts & Gahagan, 2016).

### Strengths and limitations

Our comprehensive analysis of the clustering of syndemic ill-health outcomes, alongside the pathogenic context that is driving these, and the potential protective effective of community assets is an original contribution to the field and provides new empirical evidence across five high-income countries from which data on syndemics was previously limited. Despite the geographical diversity, the online nature of the survey and the recruitment strategy mean that the sample and data cannot be legitimately claimed as representative of all GBMSM in these countries and the results should be interpreted within this context. Future triangulation of our results with those from population-based studies would maximise the evidence base. In SMMASH2, we found higher rates of concurrency of syndemic health outcomes than found in Natsal-3, for example, which found 8.4% of GBMSM reported all of the mental, physical and sexual health outcomes they included (Mercer et al., 2016). However, the small numbers of GBMSM in Natsal-3 precluded further analysis of the factors associated with this concurrency; a gap which our study is able to fill.

The difference in the prevalence of syndemic ill-health between the two samples is notable and may be explained by the absence of a measure of sexual function in Sex Now. To our knowledge, there are no data available on this for GBMSM in Canada and it is worthy of further exploration. Cross-national comparison of data on GBMSM is often limited by variations in countries’ behavioural surveillance data and our research would be strengthened by greater efforts to align these. The country-level differences demonstrate the need for wider macro-social analysis of pathogenic and salutogenic factors at the population-level, and to account for different social contexts, and the significance of these for the communities in question, in the development of complex interventions (Craig et al., 2008).

As is the case with all such studies, we are reliant on self-report data sampled online and the potential biases that this entails (see Frankis, Flowers, McDaid, & Bourne, 2018 for further discussion), although the anonymous and self-complete nature of the surveys will have limited social desirability bias. The Sex Now survey protocol only allowed fully-completed surveys to be used for research purposes. SMMASH2 allowed participants to miss any items or sections, because some very sensitive issues were covered (e.g. sexual abuse). Neither survey protocols permitted participants to return to and finish partially completed surveys which may have led to under or over-reporting of syndemic conditions. It is also worth noting that in SMMASH2 there were missing data across some of the variables of interest. In order to maximise the data available for analysis, clustering analyses and initial bivariate analyses were performed on an available case basis with the 2971 GBMSM with syndemic outcome data, but further restricted to a complete case analysis of 1715 GBMSM with data on the variables identified as significant in bivariate analysis for the regression models. This has allowed us to maximise the data available for the identification of syndemic clusters in an attempt to minimise bias through the loss of data. While the final regression sample is therefore ‘different’ from the bivariate sample, it is still sufficient for the analysis, and was the most reasonable of all the scenarios available to us. Issues of missing data and the likelihood that those with the greatest disease burden seem most likely not to participate in cross-sectional research, suggest that we have likely underestimated levels of syndemics among our populations. However, comparison of those excluded with those included in the regression indicated no significant differences in the prevalence of syndemic in the two groups, however this remains a potential source of bias in the results. Finally, these are cross-sectional data and causality cannot be assessed, but our findings present a clear way forward for future research, policy and practice.

### Implications for policy, practice and future research

Syndemics theory is differentiated from other theoretical health models by the inclusion of pathogenic social context at its very core. Understanding this is particularly important if we are to develop interventions to counter syndemics, and to move beyond a primarily medical approach to multi-morbidities (The Academy of Medical Sciences, 2018), to focus on the underlying, upstream determinants of ill-health. Significant levels of perceived and experienced stigma and discrimination are reported among GBMSM (Brown et al., 2017; Pachankis et al., 2017); (Government Equalities Office, 2018). However, societal and community norms have changed markedly over the last two decades (Holt, 2011; Rosser, West, & Weinmeyer, 2008), and the countries included here have all witnessed significant steps to increase (legislative) sexual and gender equality. The lack of association between stigma and discrimination and social isolation and syndemic ill-health in SMMASH2 (once other socio-demographics and individual risk behaviours were controlled for) could suggest that the experience of stigma and discrimination may not be as pertinent in these men's lives as financial hardship is in a time of ongoing austerity and financial uncertainty. We could hypothesise that the more proximate individual-level risk factors for poor ill-health are themselves on the causal pathway between upstream stigma and social isolation and downstream syndemic ill-health and this is worthy of further research. Our analysis suggests that it is important to investigate and moderate macro-social elements that distinguish the national contexts included within the study; it asks which structural elements, relating to syndemics, are amenable to change? How can we directly challenge and remove stigma and homophobia? How can the negative consequences of financial hardship be ameliorated?

Answering these questions also requires different approaches to analysing data. As suggested by Tsai et al., we need to move beyond analysis of individual risk factors to understand the complexity of syndemics better (Tsai et al., 2017). Most recently, Tsai has advocated for less individualised methods, the use of multi-level models encapsulating mixed methods approaches, and taking advantage of advances in network science and agent-based modelling (Tsai, 2018). We wholeheartedly agree. Our finding of the association between financial hardship and syndemic ill-health points to a potential influence from the broader political and economic environment in which our participants lived, but further analysis is beyond the limits of the data presented here. Future research using population-based samples and linking to administrative data sources could enlighten this and add greatly to the field. Furthermore, using network science and agent-based models to understand how, not just if, the multitude of aspects of the syndemic are correlated would greatly aid the development and evaluation of interventions (Tsai, 2018).

Our findings suggest that much can be learned from a deeper exploration of strengths and assets; how can these be maximised and moderated within an exploration of aspiration-based public health to reduce syndemic ill-health? It points to a theoretical mechanism by which salutogenic approaches to health improvement could function and new strategies for working with communities to understand and model the proposed process of change are required. Definitions and measurement of resilience among sexual minorities continue to be contested and thought inadequate at considering the intersection between the individual, social and structural inequalities faced (Colpitts & Gahagan, 2016), while few studies have examined the intersection of resilience at the individual-, peer- and community-level (de Lira & de Morais, 2017). This does not dispute that there are strengths and assets within communities and if we can better understand these (and in particular how some can resist the same challenges to health when others cannot) (Marsh, Schroeder, Dearden, Sternin, & Sternin, 2004), we can capitalise on these to develop interventions to counter syndemics. Formative qualitative research is required to map out these to fully understand what it is within social relationships and networks, and the upstream, distal, shared and overlapping determinants of ill-health that these networks are grounded in, that could and should be capitalised on in population health interventions (Berkman, Glass, Brissette, & Seeman, 2000).

Given the caveats of our diverse settings, methods and measures, our exploratory analysis is still suggestive of steps forwards with intervening to reduce syndemics in future generations of GBMSM. It has been suggested that interventions that only target individual health or risk behaviours are unlikely to solve the complex, multi-faceted, interrelated issues that come together in this population, with multiple health-related consequences (Stall et al., 2015), but much literature still focuses on single health issues (Herbst et al., 2007; Sherr, Clucas, Harding, Sibley, & Catalan, 2011; Wray et al., 2016). Conceptualising syndemic health inequalities within a complex adaptive systems model (Rutter et al., 2017) could enable consideration of not just the development of syndemics within individuals, but also how population-level factors could increase risk or foster resilience over time. While complex interventions could be more efficient and synergistic than multiple, separate interventions that may duplicate effort or be competitive and even counterproductive, the single health issue intervention should not be entirely discounted (Tsai et al., 2017). We would argue that we need both and a multidisciplinary partnership approach would be best instigated to achieve this. A system-level intervention would improve the future health of GBMSM and be cost-effective in the longer term, but we still need more downstream and condition-specific interventions (e.g. PrEP) to counter the ill-health experiences caused by earlier, and current, experiences of pathogenic social contexts. Such a combined approach would encapsulate the preventative complex interventions that are needed to sort out problems in the future alongside stratified, targeted approaches for those who are currently ‘victims’ of syndemics (addressing both mental and sexual health with more reparative intensive interventions). Over time, we would expect the former to mean that less of the latter are required.

## Conclusions

Our work presents new understanding of syndemics and provide new insights into how to intervene. Much of what we have drawn on could relate to other marginalised groups affected by health inequalities and is a useful addition to the field as we seek new means of understanding and countering multi-morbidities (The Academy of Medical Sciences, 2018). It represents a starting point for future research to extend this work, and develop new population health interventions (Chaudoir, Wang, & Pachankis, 2017). Experiences of health are driven by the profound experience of social inequalities and our efforts to improve health need to be salient of and address these inequalities. Our efforts need to be grounded in understanding of the lived experience of the most marginalised, working with communities to identify the means of countering syndemics. While many questions remain, addressing these will help us to understand the complexity of syndemics and the potential to combat them with salutogenic, strengths-based interventions.

## Funding

LMcD and PF are funded by the United Kingdom Medical Research Council (MRC) and Scottish Government Chief Scientist Office (CSO) at the MRC/CSO Social & Public Health Sciences Unit, University of Glasgow fund LMcD and PF (MC_UU_12017/11, SPHSU11; MC_UU_12017/12, SPHSU12). JF and KMK are funded by Glasgow Caledonian University. The SMMASH2 study was partly funded from a grant from NHS Greater Glasgow and Clyde and NHS Lothian, United Kingdom. Postdoctoral funding for OF was provided by Movember Canada (Grant # 11R18296), the Canadian Institutes for Health Research (Grant # 11R06913) and the Michael Smith Foundation for Health Research in Canada (Grant # 17945). MG is funded by the Provincial Health Services Authority, British Columbia, Canada. The Sex Now 2014-15 survey was partly funded by a grant from the Vancouver Foundation and the Public Health Agency of Canada. The funders had no role in the preparation or submission of the manuscript, and the views expressed are those of the authors alone.

## Authors’ contributions

JF, LMcD and PF designed the SMMASH2 study and OF and MG were part of the team who designed the Sex Now study. LMcD devised the paper and prepared the first draft. Data were analysed by KM and OF, with input from all authors into interpretation of the results. All authors contributed to drafting and revising the manuscript and approved the final version.

## Ethical statement

Ethical approval for Survey 1 was granted by Glasgow Caledonian University School of Health and Life Sciences Ethics Subcommittee (HLS id: HLS/NCH/15/26) and consent assumed by survey participation. The independent ethics board of the Community-Based Research Centre for Gay Men's Health reviewed and approved Survey 2 and participants indicated informed consent by clicking on a button before accessing the survey questions.

## Declaration of competing interest

None.
