# Supplementary material for: Informing theoretical development of salutogenic, asset-based health improvement to reduce syndemics among gay, bisexual and other men who have sex with men: Empirical evidence from secondary analysis of multi-national, online cross-sectional surveys
Source: SSM Popul Health. 2019 Nov 27;10:100519. doi: 10.1016/j.ssmph.2019.100519 (PMC6911981; doi:10.1016/j.ssmph.2019.100519)
Supplement: Multimedia component 1 [file mmc1.docx]

Online Supplementary File 1: SMMASH2 and Sex Now Study measures

|  | **SMMASH2** | **Sex Now** |
| --- | --- | --- |
| **Measure** | **Questionnaire variable** | **Questionnaire variable** |
| *Socio-demographics* |  |  |
| Country of residence | Country resident in at time of completing the questionnaire:  Scotland  Wales  Northern Ireland  Republic of Ireland | N/A |
| Age | Age recoded as (years):  16-25  26-35  36-45  46 and over | Age recoded as (years):  18-25  26-35  36-45  46 and over |
| Relationship status | Current partnership status:  Single  Regular Male Partner  Civil Partnership/Married  Regular female partner | Current relationship status:  Single  Partnered or married to a man  Partnered or married to a woman  Separated, divorced, other |
| Education | Highest qualification level attained:  None  Secondary  Degree  Postgraduate | Highest level of education completed:  Some or High School completed  Some college/University  College or University degree  Graduate degree |
| Employment | Current employment status:  Employed  Unemployed  Retired  Student  Long-term sick/carer | Current Employment:  Employed  Self-employed  Student  Retired  Unemployed  Unable to work |
| Ethnicity | Ethnicity:  White  Non-white | Ethnic/cultural origins: White  Indigenous  Non-white |
| Sexual orientation | Sexual orientation:  Gay  Bisexual  Straight | Sexual identity:  Gay  Bisexual  Straight/other |
| Financial worries | Current financial worries:  Never/occasionally  Sometimes/Most of the time/All of the time | Income in the last year:  Under CAD $30,000  CAD $ 30,000 – 59,999  CAD $60,000 or + |
| Recent HIV testing behaviour | Testing recoded as:  3 monthly  6 monthly  Annually  Sporadic  Don’t test as HIV positive  Don’t text and unknown status | HIV test in the past 12 months:  Yes  No |
| *Syndemic health outcomes* |  |  |
| Sexual health | Any STI diagnosis in the last 12 months | Any STI diagnosis in the last 12 months |
|  | Experience of interpersonal partner violence (verbal, emotional or physical abuse) in the past 12 months | Partner violence:  Ever mistreated (verbal, emotional or physical abuse) by a sex partner in the last 12 months |
|  | Low level of sexual satisfaction in the past 12 months |  |
| Physical health | A long-term health condition or disability  (incl. developmental disorder, learning difficulty or disability, blindness or partial sight loss, deafness or partial hearing loss, physical disability, long-term illness of disease other than HIV) | Ever diagnosed with a chronic condition (incl. high blood pressure, highcholesterol, diabetes, cardiovascular disease, cancer) |
|  | Unhealthy BMI (<18 or >25) calculated from self-reported height and weight | General health (physical, mental, social wellbeing) self-assessment  Poor / fair  Good  Very good / excellent |
| Mental health | A mental health diagnosis by a doctor  (incl. depression, anxiety, OCD, phobia, eating disorder, post-traumatic stress, bipolar disorder, schizophrenia, psychotic illness) | Ever considered or attempted suicide |
|  | Current receipt of medication for a mental health condition | Discussing issues with mental health (anxiety, depression) with a health professional in the past 12 months |
|  | Affected by a mental health condition on the past 12 months  (as above) | Self-medication for anxiety or depression without a prescription in the last 12 months |
|  | GAD-7^1^ score ≥10, indicative of moderate/severe anxiety |  |
|  | PHQ-9^2^ score ≥15, indicative of moderately severe/severe depression |  |
| *Syndemic health behaviours/drivers* |  |  |
| Sexual health | Condomless anal intercourse (CAI) with higher risk for HIV infection (CAI with≥2, casual and/or with unknown/discordant partners in the previous 12 months | condomless sex with 2+ partners of unknown HIV status in the past 12 months |
| Mental health | None included | None included |
| Physical health | Current smoking  Current vaping  Binge drinking in last 12 months (8+ drinks on one occasion once a month or more)  Party drug use (Cocaine, Ecstasy, Crystal methamphetamine, GHB, Ketamine) in last 4 weeks  Other drug use (Poppers, Legal Highs, Cannabis) in last 4 weeks  Physical inactivity (not reporting at least 150 minutes of moderate activity and strength exercises on two or more days per week, or at least 75 minutes of vigorous aerobic activity and strength exercises on two or more days per week)^3^ | Tobacco use  Binge drinking (5+ drinks in one sitting) in the last 12 months at least once a week, every week  Party drug use (Cocaine, Ecstasy, Crystal methamphetamine, GHB, Ketamine) in last 12 months  Other drug use (Poppers, Marijuana, Mephedrone) in last 12 months |
| *Social isolation* |  |  |
| Preferred relationship | Preferred relationship status is different from current status:  Current  Different  Don’t mind |  |
| Social participation (on scene) | Number of times out on the gay scene in the last month(five point scale, analysed as a continuous variable)  4-5x per week  1-2x per week  2-3x per month  Once per month or less  Never | Satisfaction with meeting guys at:  Social groups and events  Gay bars and clubs  Five point scale coded as:  Unsatisfied / very unsatisfied  Satisfied / very satisfied  Not sure / NA |
| Social participation (online) | Frequency of SNS use  Derived from a single item ‘ How often do you use Gay Social Networking WEBSITES (like Gaydar, Fitlads, Recon, Squirt etc.)?’ Coded for higher scores indicating more frequent use | Satisfaction with meeting guys at:  At internet sites  On social location apps  Five point scale coded as:  Unsatisfied / very unsatisfied  Satisfied / very satisfied  Not sure / NA |
| Self-reported outness | Five point scale from ‘out to everyone’ to ‘not out to anyone’ | Who knows about your sexuality:  Friends  Family  School/college/university  Workplace  Civic, community activities  (coded as dichotomous out to everyone vs not) |
| Experience of stigma and discrimination due to sexuality | Personalised Gay Stigma Scale consists of 9 items which measure awareness of societal attitudes about gay people and the potential social consequences related to being gay.^4^ | Experienced discrimination in the last 12 months in at least one of the following settings/situations:   - family events - renting an apartment or house - workplace - healthcare - recreational setting - justice system - Canadian border - borders of other countries |
|  |  | Worry of anti-gay stigma in the following settings:   - family events - renting an apartment or house - workplace - healthcare - recreational setting - justice system - Canadian border - borders of other countries   Five point scale coded from 1(low worry) to 5 (high).  Coded as dichotomous high worry (4 or 5) in at least one setting. |
|  |  |  |
| *Assets* |  |  |
| Resilience | Sense of coherence 13 item ‘Orientation to Life’ scale (total score for all 13 items used in the analysis - higher scores indicating greater sense of coherence). Assessing an individual’s ability to understand the world they live in, and make use of internal and external resources towards making health promoting decisions, with a higher score indicative of greater resilience.^5^ | Aspirations – likelihood of achieving:  The quality of life that you want  Enough money to live as you wish  Own property  Marry another man  Parent a child or children  (Five point scale from very unlikely to already have) |
|  | Emotional competence is the ability to understand and regulate emotions skillfully to help improve your wellbeing. This was assessed by the 30 item ‘Trait-Emotional Intelligence Questionnaire – Short Form’ scale. The total score across all 30 items was used in the analysis, with higher scores indicating greater emotional competence.^6^ |  |
| Community engagement |  | Free time spent with other gay or bisexual men  <50%  50%  >50% |

^1^ Spitzer, R.L., Kroenke, K., Williams, J.B. & Lowe, B. 2006. A brief measure for assessing generalized anxiety disorder: the GAD-7. Arch Intern Med, vol. 166, no. 10, pp. 1092-7.

^2^ Kroenke, K., Spitzer, R.L. & Williams, J.B. 2001. The PHQ-9: validity of a brief depression severity measure. J Gen Intern Med, vol. 16, no. 9, pp. 606-13.

^3^ In line with the UK Government recommendations for physical activity.

^4^ Frost, D., Parsons, J. & Nanin, J. 2007. Stigma, concealment and symptoms of depression as explanations for sexually transmitted infections among gay men. Journal of Health Psychology, vol. 12, no. 4, pp. 636-640.

^5^ Naaldenberg, J., Tobi, H., van den Esker, F., & Vaandrager, L. (2011). Psychometric properties of the OLQ-13 scale to measure Sense of Coherence in a community-dwelling older population. *Health Qual Life Outcomes,* 9, 37

^6^ Petrides, K.V. & Furnham, A. 2003. Trait emotional intelligence: Behavioural validation in two studies of emotion recognition and reactivity to mood induction. European Journal of Personality, vol. 17, no. 1, pp. 39-57; Petrides, K.V. & Furnham, A. 2006. The role of trait emotional intelligence in a gender-specific model of organizational variables. Journal of Applied Social Psychology, vol. 36, no. 2, pp.552-569.
