# Supplementary material for: Informing theoretical development of salutogenic, asset-based health improvement to reduce syndemics among gay, bisexual and other men who have sex with men: Empirical evidence from secondary analysis of multi-national, online cross-sectional surveys
Source: SSM Popul Health. 2019 Nov 27;10:100519. doi: 10.1016/j.ssmph.2019.100519 (PMC6911981; doi:10.1016/j.ssmph.2019.100519)
Supplement: Multimedia component 2 [file mmc2.docx]

**Online Supplementary File 3: Bivariate associations of syndemic ill health (the experience of 2 or more negative health outcomes) with socio-deomographics, syndemic health drivers, social isolation and stigma, and community assets: %, n, and Chi-square results**

|  | SMMASH2 | | | | Sex Now | | | |
| --- | --- | --- | --- | --- | --- | --- | --- | --- |
|  | Syndemic ill health | | | | Syndemic ill health | | | |
|  | No  % (n) | Yes  % (n) | χ^2^ | P-value | No  % (n) | Yes  % (n) | χ^2^ | P-value |
| ***Socio-demographics*** |  |  |  |  |  |  |  |  |
| **Age** |  |  | 2.751 | .432 |  |  | 83.627 | .000 |
| 16 – 25 | 24.5 (116) | 75.5 (357) |  |  | 78.3  (906) | 21.7  (251) |  |  |
| 26 – 35 | 25.9 (159) | 74.1 (455) |  |  | 75.2  (1293) | 24.8  (426) |  |  |
| 36 – 45 | 22.3 (144) | 77.7 (502) |  |  | 69.7  (946) | 30.3  (412) |  |  |
| 46 and over | 23.1 (237) | 76.9 (791) |  |  | 66.3  (2374) | 33.7  (1208) |  |  |
| **Country** |  |  | 4.906 | .179 |  |  |  |  |
| Scotland | 24.3 (328) | 75.7 (1020) |  |  |  |  |  |  |
| Wales | 20.5 (92) | 79.5 (356) |  |  |  |  |  |  |
| Northern Ireland | 21.0 (45) | 79.0 (169) |  |  |  |  |  |  |
| Republic of Ireland | 25.5 (193) | 74.5 (565) |  |  |  |  |  |  |
| **Education level** |  |  | 8.578 | .035 |  |  | 2.834 | .420 |
| None | 14.3  (6) | 85.7  (35) |  |  | 69.2 (769) | 30.8  (342) |  |  |
| Secondary | 21.2 (207) | 78.8 (768) |  |  | 69.6  (1227) | 30.4  (537) |  |  |
| Degree | 25.8 (325) | 74.2 (936) |  |  | 71.2  (2640) | 28.8  (1070) |  |  |
| Postgraduate | 24.8 (108) | 75.2 (328) |  |  | 71.3  (918) | 28.7  (369) |  |  |
| **Ethnicity** |  |  | 2.919 | .088 |  |  | 12.930 | .002 |
| White | 23.5 (631) | 76.5 (2055) |  |  | 71.2  (4532) | 28.8 (1833) |  |  |
| Non-white | 32.0 (24) | 68.0  (51) |  |  | 69.5  (796) | 30.5  (350) |  |  |
| Indigenous |  |  |  |  | 62.6  (226) | 37.4  (135) |  |  |
| **Sexual orientation** |  |  | 15.638 | .001 |  |  | 20.842 | .000 |
| Gay | 22.2 (492) | 77.8 (1727) |  |  | 69.3  (3509) | 30.7  (1553) |  |  |
| Bisexual | 29.0 (145) | 71.0 (355) |  |  | 75.1  (1253) | 24.9  (416) |  |  |
| Straight | 43.5 (10) | 56.5  (13) |  |  | 69.4  (792) | 30.6  (349) |  |  |
| **Relationship Status** |  |  | 11.244 | .010 |  |  |  |  |
| Single | 21.8 (364) | 78.2 (1305) |  |  |  |  |  |  |
| Regular Male Partner | 26.5 (158) | 73.5 (439) |  |  |  |  |  |  |
| Civil Partnership/Married (to a man) | 24.2 (54) | 75.8 (169) |  |  |  |  |  |  |
| Regular female partner | 29.7 (80) | 70.3 (189) |  |  |  |  |  |  |
| **Relationship status (Sex Now)** |  |  |  |  |  |  | 73.021 | .000 |
| Single |  |  |  |  | 68.0  (2395) | 32.0  (1126) |  |  |
| Partnered or married to a man |  |  |  |  | 72.3  (1721) | 27.7  (661) |  |  |
| Partnered or married to a woman |  |  |  |  | 78.2  (1048) | 21.8  (293) |  |  |
| Separated, widowed, other |  |  |  |  | 62.1 (390) | 37.9  (238) |  |  |
| **Employment Status** |  |  | 38.837 | .000 |  |  | 137.040 | .000 |
| Employed | 25.4 (516) | 74.6 (1514) |  |  | 72.5  (5180) | 27.5 (1968) |  |  |
| Unemployed  (or unable to work in Sex Now) | 10.5 (17) | 89.5 (145) |  |  | 51.7  (374) | 48.3  (350) |  |  |
| Retired | 23.8 (43) | 76.2 (138) |  |  |  |  |  |  |
| Student | 26.0 (67) | 74.0 (191) |  |  |  |  |  |  |
| Long-term sick/carer | 6.2  (7) | 93.8 (106) |  |  |  |  |  |  |
| **HIV Status and Testing** |  |  | 16.434 | .006 |  |  |  |  |
| 3 monthly | 21.5 (41) | 78.5 (150) |  |  |  |  |  |  |
| 6 monthly | 21.6 (82) | 78.4 (297) |  |  |  |  |  |  |
| Yearly | 23.6 (98) | 76.4 (317) |  |  |  |  |  |  |
| Sporadic | 22.0 (198) | 78.0 (702) |  |  |  |  |  |  |
| Don’t as HIV positive | 14.0 (31) | 86.0 (190) |  |  |  |  |  |  |
| Don’t test and unknown status | 27.0 (156) | 73.0 (422) |  |  |  |  |  |  |
| **HIV Status** |  |  |  |  |  |  | 145.870 | .000 |
| HIV positive |  |  |  |  | 52.5  (353) | 47.5  (320) |  |  |
| HIV negative |  |  |  |  | 70.9  (4206) | 29.1  (1727) |  |  |
| Untested |  |  |  |  | 78.6  (995) | 21.4  (271) |  |  |
| **Financial Worries** |  |  | 62.708 | .000 |  |  |  |  |
| Never/occasionally | 29.2 (459) | 70.8 (1112) |  |  |  |  |  |  |
| Sometimes/Always | 16.3 (193) | 83.7 (993) |  |  |  |  |  |  |
| **Income** |  |  |  |  |  |  | 30.151 | .000 |
| Under CAD $30,000 |  |  |  |  | 66.3  (1520) | 33.7  (773) |  |  |
| CAD $ 30,000 – 59,999 |  |  |  |  | 71.4  (1735) | 28.6  (696) |  |  |
| CAD $60,000 or + |  |  |  |  | 73.0  (2299) | 27.0  (849) |  |  |
| **Syndemic health behaviours/drivers/precursors** |  |  | 609.805 | .000 |  |  | 129.735 | .000 |
| None | 44.5 (429) | 55.5 (533) |  |  | 79.7  (1464) | 20.3  (374) |  |  |
| Sexual only | 38.7 (185) | 61.3 (293) |  |  | 68.9  (102) | 31.1  (46) |  |  |
| Physical only | 3.5  (28) | 96.5 (773) |  |  | 69.2  (3453) | 30.8  (1535) |  |  |
| Both sexual and physical | 1.4  (7) | 98.6 (496) |  |  | 59.6  (535) | 40.4  (363) |  |  |
| **Social isolation** |  |  |  |  |  |  |  |  |
| Preferred relationship |  |  | 12.214 | .002 |  |  |  |  |
| Current | 27.5 (259) | 72.5 (684) |  |  |  |  |  |  |
| Different | 22.1 (389) | 77.9 (1375) |  |  |  |  |  |  |
| Don’t mind | 14.6  (7) | 85.4  (41) |  |  |  |  |  |  |
|  | $\bar{\mathbf{x}}$ **(s.d)** | $\bar{\mathbf{x}}$ **(s.d)** | **t (df)** |  |  |  |  |  |
| Frequency of Gay scene | 1.71 (.88) | 1.73 (.90) | -.477 (2752) | .634 |  |  |  |  |
| SNS use | 2.98 (1.04) | 3.03 (1.04) | -.850 (2377) | .395 |  |  |  |  |
| Outness | 3.34 (1.55) | 3.62 (1.41) | -4.144 (1010.01) | .000 |  |  |  |  |
| Stigma/discrimination | 21.12 (5.37) | 21.45 (5.49) | -1.050 (2233) | .294 |  |  |  |  |
| Out to everyone |  |  |  |  |  |  | 40.026 | .000 |
| No |  |  |  |  | 74.7 (2253) | 25.3 (764) |  |  |
| Yes |  |  |  |  | 68.0 (3301) | 32.0 (1554) |  |  |
| **Dissatisfied with meeting men at social events** |  |  |  |  |  |  | 53.279 | .000 |
| No/unsure |  |  |  |  | 73.4  (3708) | 26.6  (1347) |  |  |
| Yes |  |  |  |  | 65.5  (1846) | 34.5  (2871) |  |  |
| **Dissatisfied with meeting men at bars** |  |  |  |  |  |  | 66.243 | .000 |
| No/unsure |  |  |  |  | 74.2 (3335) | 25.8  (1161 |  |  |
| Yes |  |  |  |  | 65.7  (2219) | 34.3  (3376) |  |  |
| **Dissatisfied with meeting men online** |  |  |  |  |  |  | 33.306 | .000 |
| No/unsure |  |  |  |  | 72.8  (3556) | 27.2  (1326) |  |  |
| Yes |  |  |  |  | 66.8  (1998) | 33.2  (992) |  |  |
| Unsure |  |  |  |  | 73.6 (999) | 26.4 (358) |  |  |
| **Dissatisfied with meeting men on apps** |  |  |  |  |  |  | 30.654 | .000 |
| No/unsure |  |  |  |  | 72.6  (3795) | 27.4  (1434) |  |  |
| Yes |  |  |  |  | 66.6  (1759) | 33.4  (884) |  |  |
| Unsure |  |  |  |  | 72.6 (2026) | 27.4 (766) |  |  |
| **Any discrimination in the last 12 months** |  |  |  |  |  |  | 146.177 | .000 |
| No |  |  |  |  | 73.3 (4806) | 26.7 (1747) |  |  |
| Yes |  |  |  |  | 56.7 (748) | 43.3 (571) |  |  |
| **High worry of discrimination** |  |  |  |  |  |  | 105.595 | .000 |
| No |  |  |  |  | 75.4 (3233) | 24.6 (1056) |  |  |
| Yes |  |  |  |  | 64.8 (2321) | 35.2 (1262) |  |  |
| **Community assets and resilience** |  |  |  |  |  |  |  |  |
| Sense of coherence total | 49.95 (11.16) | 38.78  (13.3) | 15.492 (458.833) | .000 |  |  |  |  |
| Emotional competence total | 2.74 (0.73) | 3.34  (0.90) | -12.372 (455.136) | .000 |  |  |  |  |
| **Aspirations** |  |  |  |  |  |  |  |  |
| ***Quality of life*** |  |  |  |  |  |  | 320.056 | .000 |
| Unlikely |  |  |  |  | 53.8 (981) | 46.2  (842) |  |  |
| Likely/already have |  |  |  |  | 75.6  (4573) | 24.4  (1476) |  |  |
| ***Enough money to live as you wish*** |  |  |  |  |  |  | 250.152 | .000 |
| Unlikely |  |  |  |  | 58.6 (1458) | 41.4 (1030) |  |  |
| Likely/already have |  |  |  |  | 76.1 (4096) | 23.9 (1288) |  |  |
| ***Owning property*** |  |  |  |  |  |  | 168.889 | .000 |
| Unlikely |  |  |  |  | 59.2 (1203) | 40.8 (828) |  |  |
| Likely/already have |  |  |  |  | 74.5 (4351) | 25.5 (1490) |  |  |
| **Free time spent with other gay and bi men** |  |  |  |  |  |  | 2.016 | .156 |
| Less than 50% |  |  |  |  | 71.0 (3904) | 29.0 (1592) |  |  |
| More than 50% |  |  |  |  | 69.4 (1650) | 30.6 (726) |  |  |
