# Supplementary material for: Informing theoretical development of salutogenic, asset-based health improvement to reduce syndemics among gay, bisexual and other men who have sex with men: Empirical evidence from secondary analysis of multi-national, online cross-sectional surveys
Source: SSM Popul Health. 2019 Nov 27;10:100519. doi: 10.1016/j.ssmph.2019.100519 (PMC6911981; doi:10.1016/j.ssmph.2019.100519)
Supplement: Multimedia component 3 [file mmc3.docx]

**Online supplementary file 2: Sexual, mental and physical health outcomes in SMMASH2 and Sex Now**

|  | SMMASH2 | | | | Sex Now | | | |
| --- | --- | --- | --- | --- | --- | --- | --- | --- |
|  | Yes |  | No |  | Yes |  | No |  |
|  | N | % | N | % | N | % | N | % |
| ***Sexual health*** |  |  |  |  |  |  |  |  |
| Any STI diagnosis in the past 12 months | 268 | 9.0 | 2710 | 91.0 | 1171 | 14.9 | 6701 | 85.1 |
| Partner violence or abuse in the past 12 months | 489 | 16.5 | 2475 | 83.5 | 1043 | 13.2 | 6829 | 86.8 |
| Sexual dissatisfaction in the past 12 months | 2286 | 76.9 | 687 | 23.1 | - | - | - | - |
| ***Mental health*** |  |  |  |  |  |  |  |  |
| Mental health diagnosis | 785 | 26.4 | 2188 | 73.6 | - | - | - | - |
| Current medication for mental health problem | 392 | 13.2 | 2578 | 86.8 | - | - | - | - |
| Affected by a mental health problem in past 12 months | 860 | 28.9 | 2116 | 71.1 | - | - | - | - |
| GAD-7 score ≥10, indicative of moderate/severe anxiety | 459 | 15.4 | 2522 | 84.6 | - | - | - | - |
| PHQ-9 score ≥15, indicative of moderately severe/severe depression | 1300 | 43.8 | 1668 | 56.2 | - | - | - | - |
| Considered or attempted suicide in the past 12 months | - | - | - | - | 1480 | 18.8 | 6392 | 81.2 |
| Discussed anxiety or depression with a health care provider in the past 12 months | - | - | - | - | 2358 | 30.0 | 5514 | 70.0 |
| Self-medication for anxiety in the past 12 months | - | - | - | - | 640 | 8.1 | 7232 | 91.9 |
| ***Physical health*** |  |  |  |  |  |  |  |  |
| Chronic or long-term physical health condition | 922 | 31.0 | 2052 | 69.0 | 2716 | 34.5 | 5156 | 65.5 |
| Unhealthy BMI (less than 18 or greater than 25) | 1303 | 43.9 | 1665 | 56.1 |  |  |  |  |
| Poor/fair self-rated health | - | - | - | - | 744 | 9.5 | 7128 | 90.5 |
